# Construction and Validation of Angiogenesis-Related Prognostic Risk Signature to Facilitate Survival Prediction and Biomarker Excavation of Breast Cancer Patients

**DOI:** 10.1155/2022/1525245

**Published:** 2022-04-20

**Authors:** Yingkun Xu, Yang Peng, Meiying Shen, Li Liu, Jinwei Lei, Shun Gao, Yuan Wang, Ailin Lan, Han Li, Shengchun Liu

**Affiliations:** Department of Endocrine and Breast Surgery, The First Affiliated Hospital of Chongqing Medical University, Chongqing, 400042, China

## Abstract

This study is aimed at exploring the potential mechanism of angiogenesis, a biological process-related gene in breast cancer (BRCA), and constructing a risk model related to the prognosis of BRCA patients. We used multiple bioinformatics databases and multiple bioinformatics analysis methods to complete our exploration in this research. First, we use the RNA-seq transcriptome data in the TCGA database to conduct a preliminary screening of angiogenesis-related genes through univariate Cox curve analysis and then use LASSO regression curve analysis for secondary screening. We successfully established a risk model consisting of seven angiogenesis-related genes in BRCA. The results of ROC curve analysis show that the risk model has good prediction accuracy. We can successfully divide BRCA patients into the high-risk and low-risk groups with significant prognostic differences based on this risk model. In addition, we used angiogenesis-related genes to perform cluster analysis in BRCA patients and successfully divided BRCA patients into three clusters with significant prognostic differences, namely, cluster 1, cluster 2, and cluster 3. Subsequently, we combined the clinical-pathological data for correlation analysis, and there is a significant correlation between the risk model and the patient's T and stage. Multivariate Cox regression curve analysis showed that the age of BRCA patients and the risk score of the risk model could be used as independent risk factors in the progression of BRCA. In particular, based on this angiogenesis-related risk model, we have drawn a matching nomogram that can predict the 5-, 7-, and 10-year overall survival rates of BRCA patients. Subsequently, we performed a series of pan-cancer analyses of CNV, SNV, OS, methylation, and immune infiltration for this risk model gene and used GDSC data to explore drug sensitivity. Subsequently, to gain insight into the protein expression of these risk model genes in BRCA, we used the immunohistochemical data in the THPA database for verification. The results showed that the protein expressions of IL18, RUNX1, SCG2, and THY1 molecules in BRCA tissues were significantly higher than those in normal breast tissues, while the protein expressions of PF4 and TNFSF12 molecules in BRCA tissues were significantly lower than those in normal breast tissues. Finally, we conducted multiple GSEA analyses to explore the biological pathways these risk model genes can cross in cancer progression. In summary, we believe that this study can provide valuable data and clues for future studies on angiogenesis in BRCA.

## 1. Introduction

Breast cancer (BRCA) is the most common malignant tumor that seriously endangers women's physical and mental health [1]. Worldwide, the number and incidence of BRCA have increased rapidly, and it has now surpassed lung cancer to become the world's largest tumor type [2], although early diagnosis and early treatment nowadays have significantly improved the curative effect of breast cancer [3, 4]. However, postoperative recurrence and metastasis of BRCA are the leading causes of BRCA death, and they have now become the focus of BRCA treatment [5]. Our understanding of the pathogenesis and process of breast cancer is still in its infancy [6]. Therefore, it is necessary to continuously explore BRCA recurrence and metastasis molecular mechanisms and look for potential intervention targets.

Tumor angiogenesis is when abnormal proliferation, mainly capillary blood vessels, is generated based on original blood vessels, and blood circulation is established in tumor tissues [7]. The structure and function of new capillaries in tumor tissues are very different from normal tissues. Compared with normal blood vessels, tumor neovascularization has the characteristics of an extensive vascular endothelial gap, weak vessel wall, strong vascular permeability, and structural disorder [8]. Angiogenesis plays an essential role in the occurrence, development, invasion, and metastasis of BRCA, and it is also an independent prognostic factor of BRCA patients [9]. BRCA is a solid tumor, and its occurrence and development depend on angiogenesis [10]. Normal breast tissue has a loose structure and abundant lymph and blood supply. Therefore, new blood vessels are easily formed, and tumor metastasis occurs during the development of BRCA. There will be various biological and morphological changes in breast hyperplasia and precancerous lesions, including changes in the tumor microenvironment [11], among which tumor angiogenesis is the earliest [12].

Precision medicine is a new medical model formed under the background of the rapid development of modern gene sequencing and the fusion of bioinformatics and big data based on the Human Genome Project [13]. With the rise of precision medicine on a global scale, the precise diagnosis and treatment of BRCA are imminent, and traditional histopathological classification can no longer meet the needs of current BRCA research and treatment [14]. Traditional histopathological classification has been unable to meet BRCA research and treatment [15, 16]. The correct application of tumor molecular classification is the basic premise of contemporary precision medicine [17]. The molecular and histopathological classifications of BRCA can be better integrated so that clinicians can formulate effective and individualized treatment plans for BRCA patients more scientifically. Therefore, in this study, while exploring the biological significance of angiogenesis-related genes in BRCA, we used these genes to perform cluster analysis in BRCA. The results show that we successfully divided BRCA patients into three clusters with significant prognostic differences, namely cluster1, cluster2, and cluster3. We believe that these classification data will help the precise treatment of different BRCA patients in the future. In addition, this study used multiple bioinformatics databases and various bioinformatics methods to conduct in-depth research on angiogenesis-related genes in BRCA. We believe that this research can provide detailed and reliable data support for future scientific research and clinical treatment.

## 2. Materials and Methods

### 2.1. Data Acquisition

The Cancer Genome Atlas (TCGA) research network has performed a high-throughput analysis of many human tumors to find molecular aberrations at the nucleic acid, protein, and epigenetic levels [18]. In November 2021, we downloaded the gene expression, variation, and clinical information of 1,098 BRCA samples through the GDC (Genomic Data Commons) official portal of the TCGA database (https://portal.gdc.cancer.gov/). To find genes related to angiogenesis, we collected 48 angiogenesis-related genes through the GSEA (Gene Set Enrichment Analysis) database (https://www.gsea-msigdb.org/gsea/index.jsp) [19, 20]. The standard name of this gene set is ANGIOGENESIS, and the systematic name is M14493.

### 2.2. Data Processing and Analysis

This study used Perl and R language to download BRCA RNA-seq transcriptome data and clinical-pathological information from the TCGA database to process and draw graphs. First, we used the RNA-seq transcriptome data of the BRCA dataset in the TCGA database to use the “pheatmap” expansion package to draw a heat map of the expression of angiogenesis-related genes and use the “limma” expansion package to analyze the differences in the expression of angiogenesis-related genes. STRING can be used to predict the protein-protein interaction (PPI) network, which is an online database platform (https://cn.string-db.org/) [21, 22]. To explore the relationship between these angiogenesis-related molecules, we used the protein interaction data in the STRING database to draw a PPI network. After that, we performed a univariate Cox regression curve analysis of these angiogenesis-related molecules in BRCA to show the relationship between these molecules and the progress of BRCA. Subsequently, we used cluster analysis to classify BRCA patients into three clusters with significant prognostic differences. Then, we used the “glmnet” and “survival” expansion packages based on the R language to perform LASSO regression curve analysis and draw the corresponding survival curve. To verify the prediction accuracy of the risk model, we used the “survivalROC” extension package to perform ROC curve analysis. The risk model comprises seven genes, BTG1, IL18, PF4, RUNX1, SCG2, THY1, and TNFSF12. Subsequently, combined with clinicopathological data, we analyzed the correlation between the risk model and the pathological characteristics of BRCA patients. In particular, we performed univariate and multivariate Cox regression curve analyses through the “survival” and “forestplot” expansion packages. Subsequently, to facilitate clinical diagnosis and treatment in the future, we integrated various risk factors and used the “rms” expansion package to draw the corresponding nomogram. Finally, to explore the biological pathways that the risk model gene can affect in BRCA, we used the “plyr,” “ggplot2,” “grid,” and “gridExtra” expansion packages to perform a multi-GSEA analysis.

### 2.3. GEPIA Website

Gene Expression Profiling Interactive Analysis (GEPIA) is a website developed by Peking University, which can analyze the RNA-seq expression data of 9736 tumor samples and 8587 normal samples in the TCGA and GTEx projects (http://gepia.cancer-pku.cn/) [23, 24]. In this study, we used the online analysis tool on the GEPIA website to perform pan-cancer analysis on CNV and SNV of angiogenesis-related risk model genes. The results were displayed in the form of heat maps.

### 2.4. GSCA Website

Gene Set Cancer Analysis (GSCA) is a cross-over comprehensive cancer analysis database that integrates single gene analysis, multiple gene analysis, immune infiltration analysis, mutation analysis, and drug sensitivity analysis. It contains 33 types of cancer data from TCGA, ImmuCellAI, and GDSC (http://bioinfo.life.hust.edu.cn/GSCA/#/) [25, 26]. This study combined the angiogenesis-related risk model gene mRNA expression data and drug sensitivity data to perform a Pearson correlation analysis to obtain the correlation between the risk model gene mRNA expression and the drug IC50. FDR adjusts the *P* value.

### 2.5. TIMER Database

Tumor Immune Estimation Resource (TIMER) is a database that supports the analysis of tumor-infiltrating immune cell components (http://cistrome.org/TIMER/) [27, 28]. When we input the gene expression profile data of tumor samples, we can predict the composition of immune cells infiltrated in each tumor sample and support the analysis of the following six types of immune cells: B cell, CD8+ T cell, CD4+ T cell, macrophage, neutrophil, and dendritic cell. In this study, we used angiogenesis-related risk model gene mRNA expression data, combined with immune cell infiltration data in the TIMER database, to explore the relationship between risk model gene mRNA expression and immune cell infiltration in BRCA, and used R The language “pheatmap” expansion pack draws the corresponding heat map.

### 2.6. The Human Protein Atlas Database

The Human Protein Atlas (THPA) database provides information on the tissue and cell distribution of all 24,000 human proteins and is free for public inquiries (https://www.proteinatlas.org/). The Swedish Knut & Alice Wallenberg Foundation, which created this database, uses special antibodies and immunohistochemical techniques to examine each protein in 48 normal human tissues, 20 types of tumor tissues, 47 cell lines, and 12 types of blood cells. The distribution and expression of the results are read and indexed by professionals [29–31]. In this study, we used the immunohistochemistry data in the THPA database to explore the expression of angiogenesis-related risk model genes in BRCA tissues and normal breast tissues.

## 3. Results

### 3.1. The Expression of Angiogenesis-Related Genes in BRCA and the Interaction of the Encoded Proteins

**Figure 1:**
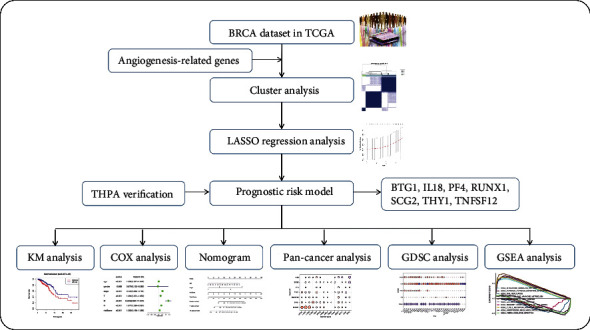
The schematic flow chart of this research.

**Figure 2:**
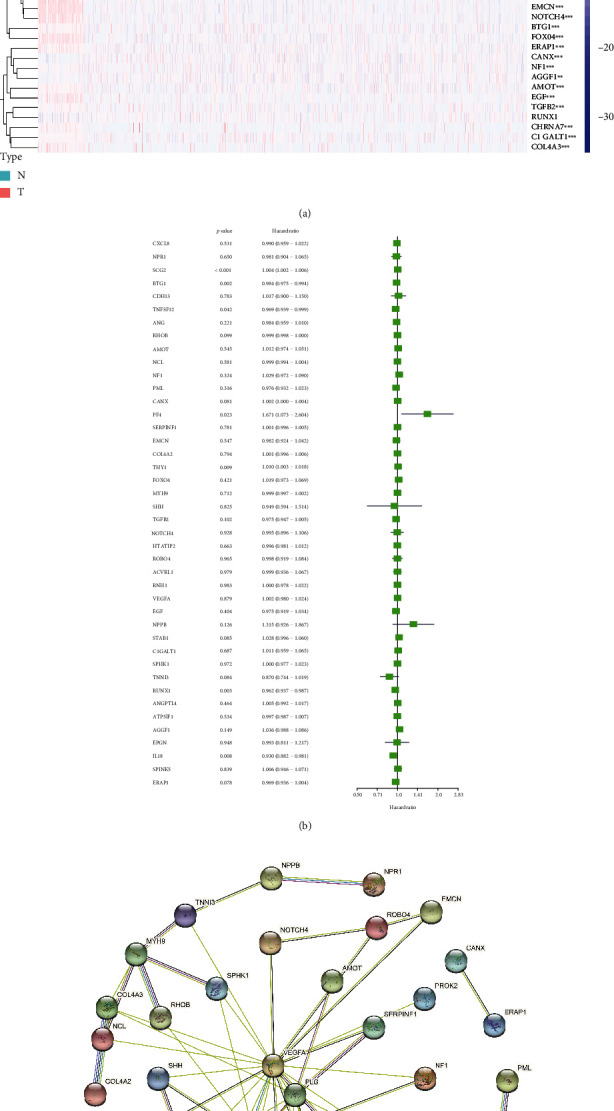
The expression of angiogenesis-related genes in BRCA and the interaction of the encoded proteins. (a) The heat map shows the expression of angiogenesis-related genes in BRCA. The redder the color, the higher the expression, and the greener the color, the lower the expression. (b) The forest plot was used to display the univariate Cox regression analysis results. (c) The PPI network was used to show the interaction between genes related to angiogenesis. ^∗^*P* < 0.05,  ^∗∗^*P* < 0.01, and^∗∗∗^*P* < 0.001.

To make our research easier to understand, we present this research's main analysis methods and steps in a flowchart (Figure 1). To understand the expression of angiogenesis-related genes in BRCA, we used the mRNA expression data in the TCGA database to draw a heat map. We find that most angiogenesis-related genes have significant differences in expression between BRCA tissues and normal breast tissues through the heat map. Among them, the expression of star molecules VEGFA, SPHK1, and SCG2 in BRCA samples was significantly higher than that in the control group. The expressions of NOTCH4, STAB1, and SERPINF1 in BRCA samples were significantly lower than those in the control group (Figure 2(a)). The results of univariate Cox analysis showed that SCG2, PF4, and THY1 played risk factors in BRCA progression, while BTG1, TNFSF12, RUNX1, and IL18 played protective factors in BRCA progression (Figure 2(b)). Then by consulting protein-protein interaction networks, we can find a strong correlation between the PF4 molecule and the CXCL8 molecule (Figure 2C). These molecules are potential targets for future BRCA prevention and control.

### 3.2. Use Angiogenesis-Related Genes to Perform Cluster Analysis in BRCA

**Figure 3:**
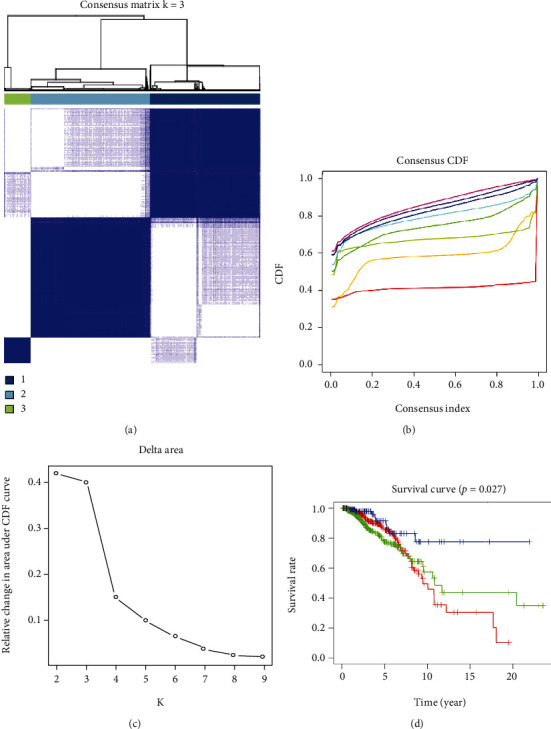
Use angiogenesis-related genes to perform cluster analysis in BRCA. (a) Consensus clustering matrix for *k* = 3. (b, c) Relative change in area under the cumulative distribution function (CDF) curve for *k* = 2–9. Consensus clustering CDF for *k* = 2–9. (d) Survival curves were drawn by three different clusters (*P* = 0.027).

In recent decades of cancer research, scientific researchers generally consider cluster analysis to provide theoretical support for precise cancer treatment. In this study, based on the TCGA database, we used the differences in the expression of these angiogenesis-related genes in BRCA patients to perform a cluster analysis. When *k* = 3, the generated consensus matrix shows a good clustering effect, and the result is verified (Figures 3(a)–3(c)). Subsequently, we developed the survival curve of BRCA patients based on the cluster analysis results (*P* = 0.027) (Figure 3(d)). Therefore, we believe that this new type of cluster classification is beneficial to future accurate clinical diagnosis and treatment.

### 3.3. Use Angiogenesis-Related Genes to Perform LASSO Regression Analysis in BRCA

**Figure 4:**
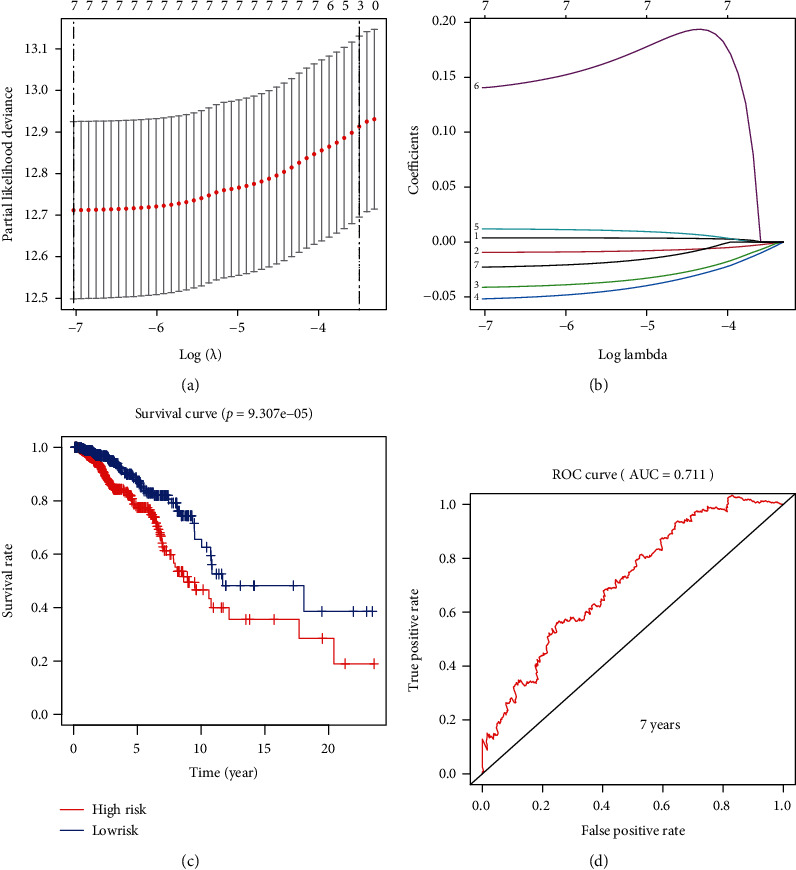
Use angiogenesis-related genes to perform LASSO regression analysis in BRCA. (a, b) LASSO regression curve analysis and cross-validation. (c) According to the best cut-off value, BRCA patients are divided into the high-risk and low-risk groups to draw the Kaplan-Meier survival curve (*P* = 9.307*e* − 05). (d) ROC curve for predicting 7-year survival time, and the value of AUC is 0.711.

To use these angiogenesis-related genes to establish a risk model in BRCA, we first performed a LASSO regression curve analysis on these angiogenesis-related genes and verified the availability of the results (Figures 4(a) and 4(b)). We successfully constructed a risk model consisting of 7 genes, including BTG1, IL18, PF4, RUNX1, SCG2, THY1, and TNFSF12. Based on this risk model, we divided BRCA patients into the high-risk and low-risk groups and drew the corresponding survival curves. The results of the survival curve show that the overall survival rate of BRCA patients in the high-risk group is significantly lower than that of BRCA patients in the low-risk group (*P* = 9.307*e* − 05) (Figure 4(c)). Finally, based on the risk model, our ROC curve analysis showed that the 7-year AUC value is 0.711 (Figure 4(d)), which implies that the risk prediction model is highly accurate.

### 3.4. Based on the Constructed Risk Model, Explore the Clinical Relevance and Draw the Nomogram

**Figure 5:**
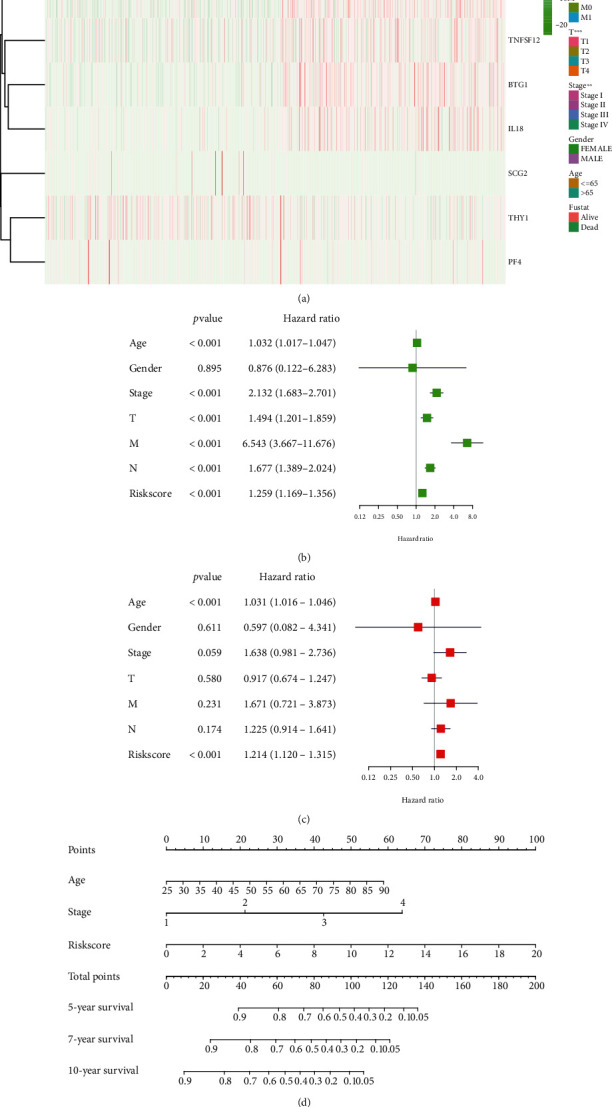
Based on the constructed risk model, explore the clinical relevance and draw the nomogram. (a) The heat map was used to show the correlation between the angiogenesis-related risk model and the clinicopathological characteristics of BRCA patients. (b) Univariate Cox regression analysis. (c) Multivariate Cox regression analysis. (d) The nomogram was used to predict the 5-, 7-, and 10-year overall survival rates of BRCA patients. ^∗^*P* < 0.05,  ^∗∗^*P* < 0.01, and^∗∗∗^*P* < 0.001.

The relationship between the risk model and clinicopathological characteristics has always been an essential direction of concern. To explore the correlation between the risk model and clinicopathological features, we conducted a correlation analysis and displayed it in the form of a heat map (Figure 5(a)). The results show that the risk model strongly correlates with the two clinicopathological characteristics of T and stage of BRCA patients.

Subsequently, we performed univariate Cox regression analysis and multivariate Cox regression analysis based on the risk model (Figures 5(b) and 5(c)). We found that the age of BRCA patients and the risk score of this risk model are independent risk factors for BRCA patients. Finally, based on the risk model, we draw a nomogram that can predict the overall survival rate of BRCA patients at 5, 7, and 10 years (Figure 5(d)).

### 3.5. Based on the Constructed Risk Model, Pan-Cancer Analysis and Sensitivity Analysis of Multiple Anticancer Drugs Were Carried Out

**Figure 6:**
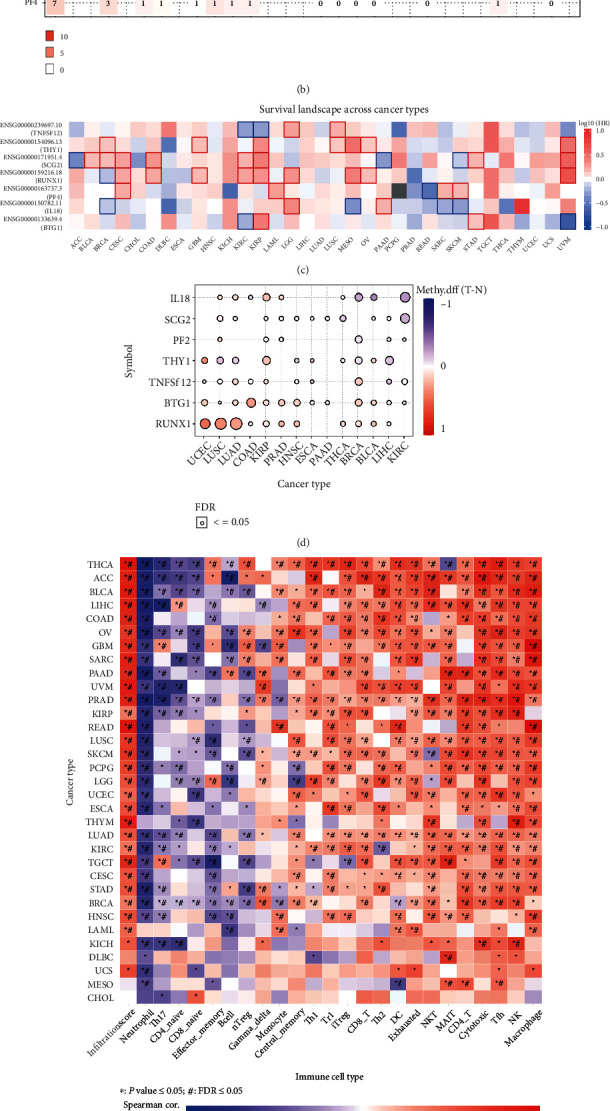
Pan-cancer analysis and sensitivity analysis of multiple anticancer drugs were carried out based on the constructed risk model. (a) The heat map shows the results of CNV analysis of seven risk model genes in pan-cancer. The light red Hete Amp represents heterozygous amplification, the light green Hete Del represents heterozygous deletion, the dark red Homo Amp represents homozygous amplification, the dark green Homo Del represents homozygous deletion, and the gray represents no CNV occurrence. (b) The heat map shows the results of SNV analysis of seven risk model genes in pan-cancer. (c) The heat map shows the OS analysis results of seven risk model genes in pan-cancer. (d) The heat map shows the methylation analysis results of seven risk model genes in various cancers. (e) The heat map shows the correlation between seven risk model genes and immune cell infiltration in pan-cancer. Red represents positive correlation and purple represents negative correlation. ^∗^P ≤ 0.05 and#FDR ≤ 0.05. (f) The heat map shows the correlation between seven risk model genes and the sensitivity of multiple anticancer drugs. Blue bubbles represent negative correlations, red bubbles represent positive correlations; the deeper the color, the higher the correlation. There is a positive correlation between bubble size and FDR significance. The black outline frame indicates FDR ≤ 0.05.

Although many studies have explored the mutations of multiple risk model genes in various cancers, the mutations in multiple cancers have not been well summarized. In addition, modern scientific research has confirmed that gene mutations may affect the overall survival rate of cancer patients [32, 33]. Therefore, this study explored and summarized the CNV, SNV, and OS of these seven risk model genes in pan-cancer. By observing the heat map showing the CNV situation, we found that RUNX1, SCG2, and BTG1 have high heterozygous amplification in various cancers, including ACC, TGCT, and UCS. However, TNFSF2, THY, and IL8 have higher heterozygous deletions in multiple cancers, including BRCA, TGCT, and SKCM (Figure 6(a)). In the results of subsequent SNV analysis, we found that RUNX1 has a higher mutation frequency in BRCA, UCEC, and BLCA, and these seven risk model genes have varying degrees of mutation frequency in UCEC and SKCM (Figure 6(b)). In analyzing the survival of these seven risk model genes in pan-cancer, we found that most genes significantly correlate with the survival of BRCA, KIRC, KIRP, and UVM patients. THY1, SCG2, and RUNX1 play risk factors in various cancer types (Figure 6(c)).

In recent years, cancer research around methylation has emerged one after another [34–36]. Therefore, we conducted a differential analysis of methylation in pan-cancer for these seven risk model genes. RUNX1 has a high methylation status in UCEC, LUSC, and LUAD, and IL18 has a low methylation status in BRCA, BLCA, and KIRC (Figure 6(d)). Then based on the ImmuCellAI database, we found that this risk model is negatively correlated with the degree of immune cell infiltration such as neutrophil, Th17, and CD8 naive and positively correlated with the degree of immune cell infiltration such as Tfh, NK, and macrophage in most cancer types (Figure 6(e)). Subsequently, to discover candidate biomarkers and valuable small molecule drugs, we used the GDSC database to closely integrate genes with clinical information and more than 750 small molecule drugs, which provided help for future experimental design and further clinical trials. The results show that the expression of IL18 and RUNX1 genes is related to the sensitivity of a variety of mainstream anticancer drugs (Figure 6(f)). We can use this correlation to provide patients with more efficient treatment options in the future.

### 3.6. For the Risk Model Genes, Explore Their Protein Expression Levels in BRCA and Normal Tissues

**Figure 7:**
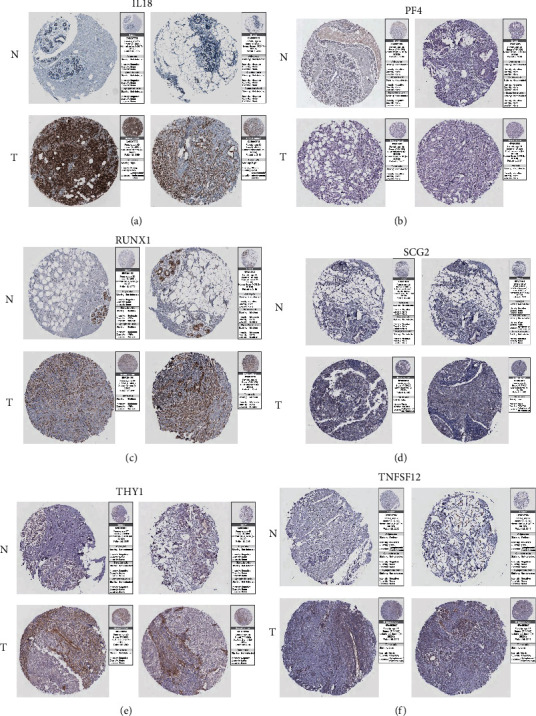
For the risk model genes, explore their protein expression levels in BRCA and normal tissues. (a) IL18, (b) PF4, (c) RUNX1, (d) SCG2, (e) THY1, and (f) TNFSF12.

To verify our previous results, we used the immunohistochemical data in the THPA database to explore the expression of these risk model genes between BRCA tissue and normal breast tissue. Here, we show the immunohistochemical images of IL18, PF4, RUNX1, SCG2, THY1, and TNFSF12 (Figures 7(a)–7(f)). These immunohistochemical results showed that the expression of IL18, RUNX1, SCG2, and THY1 molecules in normal breast tissues was significantly lower than that in BRCA tissues, while PF4 and TNFSF12 molecules showed the opposite expression. This evidence corroborates our previous findings, and our results have higher credibility.

### 3.7. For This Risk Model Genes, GSEA Analysis Was Performed in BRCA

**Figure 8:**
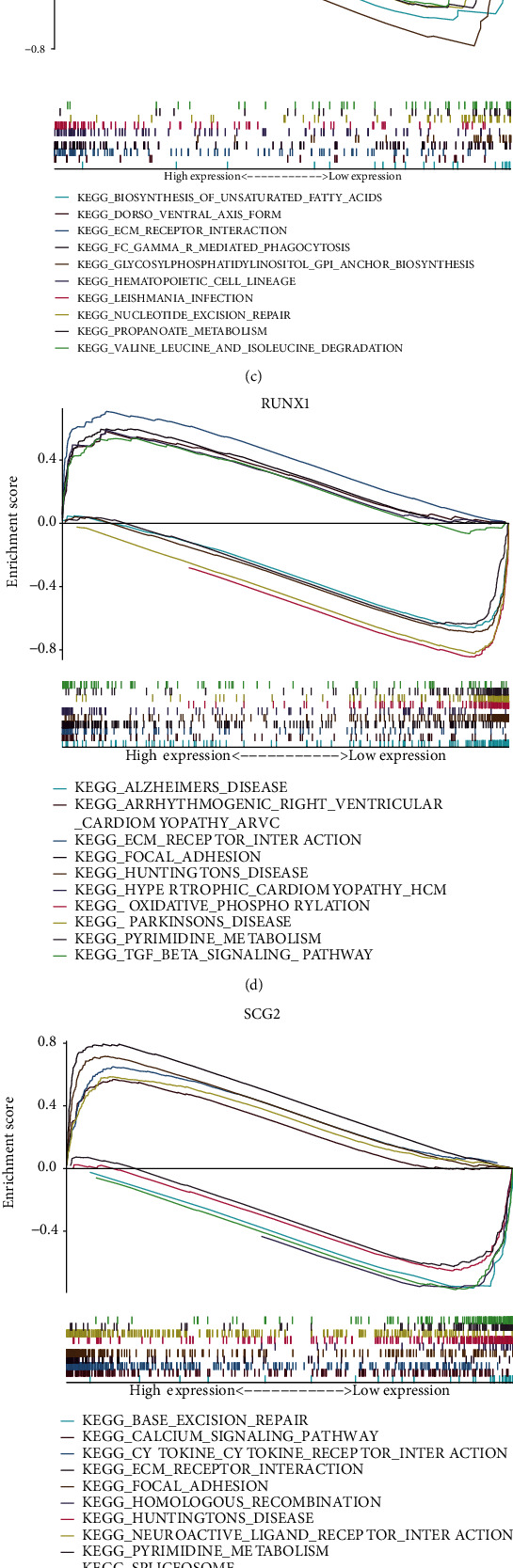
For this risk model genes, GSEA analysis was performed in BRCA. (a) BTG1, (b) IL18, (c) PF4, (d) RUNX1, (e) SCG2, (f) THY1, and (g) TNFSF12.

To deeply explore the potential biological role of these risk model genes in cancer progression, based on the TCGA database, we conducted multiple GESA analyses in BRCA for these seven risk model genes. The results show that these seven risk model genes are related to various cancer pathways in BRCA (Figures 8(a)–8(g)). For example, BTG1 and IL18 are related to abnormal activation of JAK-STAT signaling pathway. RUNX1 is related to the abnormal activation of TGF-beta signaling pathway. THY1 and TNFSF12 are related to abnormal inhibition of CELL CYCLE. Therefore, we believe that these detailed data can provide important clues for future exploration of the mechanism of these seven risk model genes in BRCA.

## 4. Discussion

In the research process of human anticancer progress, with the deepening of research, researchers gradually realized that there are many differences between the same tumors; the most fundamental difference is at the level of biomolecules. The concept of tumor molecular classification was first proposed by the National Cancer Institute in 1999. A new tumor classification system uses molecular analysis techniques to classify tumors based on molecular characteristics. In 2000, Perou et al. first proposed the molecular classification of BRCA, dividing BRCA into two groups: estrogen receptor (ER) positive and negative. The ER-positive group is called luminal type breast cancer [37]. The ER-negative group is divided into human epidermal growth factor receptor-2 (HER2) overexpression type, basal cell-like type, and normal breast-like type. Subsequently, many scholars have further confirmed and enriched the BRCA molecular typing theory through many studies and made significant progress [38–40]. Similarly, in this study, we performed a cluster analysis in BRCA using angiogenesis-related genes and could classify BRCA patients into subgroups with differences in survival. We believe this will be very helpful for precision medicine in the future.

In addition, in the past few decades, the construction of risk models around cancer-related biological processes or signaling pathway-related genes has succeeded [41–43]. Therefore, inspired by previous research, we used angiogenesis-related genes to construct a risk model for BRCA. Based on this model, we can divide BRCA patients into two groups with different overall survival rates: a high-risk group and a low-risk group. With the successful application of more and more prognostic models in clinical practice, we believe that this angiogenesis-related predictive risk model can accurately identify the risk differences of different patients [44–46]. Clinicians can use this risk difference to differentiate treatment and treatment of patients. For example, for patients in the high-risk group, the frequency of clinical therapy, testing and review can be increased, which is more conducive to patient survival.

The BTG1 gene was first identified from the chronic B lymphocytic leukemia chromosome and then isolated from lymphoblasts [47, 48]. It is located on chromosome 12q22 and can regulate cell proliferation by regulating the cell cycle [49]. Previous research reports have found that the BTG1 gene has abnormally low expression in breast cancer, gastric cancer, non-small-cell lung cancer, and pancreatic cancer tissues compared to normal tissues and is related to multiple clinical features such as lymph node metastasis, TNM (Tumor Node Metastasis) stage, and prognosis [50–53]. This shows that the BTG1 gene plays a similar role to tumor suppressor genes in the occurrence and development of various cancers and may be a potential tumor biomarker and therapeutic target. IL-8 is a proinflammatory cytokine, which plays a complex role in regulating tumor microenvironment [54] and may lead to tumor cell proliferation, survival, and chemoresistance of malignant diseases [55, 56]. High serum IL-8 expression is now associated with poor prognosis of patients with various tumors (including BRCA) [57, 58]. In BRCA, patients with high serum IL-8 levels have a worse prognosis than patients with low IL-8 levels [59, 60].

In previous studies, researchers have determined that PF4 can be used as an antiangiogenic factor to inhibit endothelial cell proliferation, migration, and angiogenesis in a variety of in vitro and in vivo models of cancer [61–64]. Among them, in the in vivo model of BRCA, upregulating PF4 can increase the expression of proapoptotic protein and downregulate the expression of antiapoptotic protein, thereby promoting cell apoptosis and achieving the effect of reducing tumor volume [65]. RUNX1 is a member of the RUNX transcription factor family. It is located at 21q22 and contains 138 amino acid Runt homologous functional regions. Existing studies have found that RUNX1 exerts a tumor suppressor effect in liver cancer and gastric cancer [66, 67], but in non-small-cell lung cancer and endometrial cancer [68, 69]. It plays a role in promoting cancer in cancer and in suppressing or promoting cancer in different subtypes of BRCA [70, 71]. As an essential transcription factor, RUNX1 mainly acts by directly or indirectly regulating signal transduction pathways such as TGF*β*, WNT, and BMP [72]. However, the three genes SCG2, THY1, and TNFSF12 have not been studied in BRCA. In the future, we need to conduct in-depth exploration to determine the potential role of these three genes in BRCA.

## 5. Conclusion

In this study, based on multiple biological information databases, we used angiogenesis-related genes to perform a series of analyses such as univariate Cox analysis, cluster analysis, LASSO regression analysis, pan-cancer analysis, and multi-GSEA analysis in BRCA and successfully constructed a predictive risk model consisting of seven genes BTG1, IL18, PF4, RUNX1, SCG2, THY1, and TNFSF12, although this study still has some shortcomings. For example, it has not been supported by single-center or multicenter clinical data. However, we believe that this research will provide many valuable clues for future scientific research. We will continue to explore the potential mechanisms of these risk model genes in BRCA progression in future scientific explorations.

## Data Availability

The data used to support the findings of this study are available from the corresponding author upon request.

## Abbreviations

BRCA: Breast cancer
TCGA: The Cancer Genome Atlas
GDC: Genomic Data Commons
GEPIA: Gene Expression Profiling Interactive Analysis
GSCA: Gene Set Cancer Analysis
TIMER: Tumor Immune Estimation Resource
THPA: The Human Protein Atlas
ImmuCellAI: Immune Cell Abundance Identifier
GDSC: Genomics of Drug Sensitivity in Cancer
LASSO: Least Absolute Shrinkage and Selection Operator
GSEA: Gene Set Enrichment Analysis
BTG1: BTG Antiproliferation Factor 1
IL18: Interleukin 18
PF4: Platelet Factor 4
RUNX1: RUNX Family Transcription Factor 1
SCG2: Secretogranin II
THY1: Thy-1 Cell Surface Antigen
TNFSF12: TNF Superfamily Member 12.
